# Non-ketotic hyperglycaemia induced occipital reflex focal seizures

**DOI:** 10.1016/j.heliyon.2023.e18355

**Published:** 2023-07-16

**Authors:** Helena Buque, Deise Catamo, Catarina Felix, Ana André, Inês Gil, Hipólito Nzwalo

**Affiliations:** a Neurological Department, Central Hospital of Maputo, Mozambique; b Faculty of Medicine and Biomedical Sciences, University of Algarve, Portugal; c Neurological Department, Algarve University Hospital Center, Portugal; d Radiology Department, Algarve University Hospital Center, Portugal; e Algarve Biomedical Sciences Research Institute, Portugal

**Keywords:** Focal seizures, Hyperglycaemic states, Transient brain signs

## Abstract

A myriad of neurological manifestations can occur in association with ketotic and non ketotic hyperglycaemic states. Contrary to diabetic coma, which is a universal complication under relatively established metabolic circumstances, the pathophysiology beyond hyperglycaemic-associated positive neurological manifestations, including seizures, remains to be elucidated. The occurrence of symptomatic focal epilepsy as a manifestation of diabetes-related hyperglycaemia is seldom reported. Herein, we present a case of focal epilepsy with alternating positive and negative neurological manifestations as the initial manifestation of diabetes-related hyperglycaemia. The electroencephalogram confirmed the diagnosis of focal occipital seizures, and the brain magnetic resonance imaging depicted the associated typical transient imaging findings in the occipital lobe. Seizures were refractory to antiepileptics, and symptomatic control was achieved after achieving normoglycemia. On follow-up, complete clinical and imaging recovery occurred. Reflex focal epilepsy in the context of hyperglycaemic states is a rare condition, and the possibility of misdiagnosis is likely high. As reported in similar cases, seizures can be resistant to antiepileptics. An important message to highlight is that seizures associated with hyperglycaemic status can be resistant to antiepileptic treatment and only cease with glycaemic control.

## Highlights

• Symptomatic focal epilepsy as a manifestation of diabetic related hyperglycaemia states is rare.
• Treatment of hyperglycemia is the main strategy to control symptomatic focal seizures in hyperglycemia states.
• Total clinical and imaging improvement is possible with adequate treatment.

## 1 Introduction

A myriad of neurological manifestations, ranging from coma to rarer positive phenomenon such as seizures, hallucinations, somatosensory symptoms, and hyperkinetic movement disorders, can occur in association with ketotic and non ketotic hyperglycaemic states [1]. Contrary to diabetic coma, which is a universal complication under relatively established metabolic circumstances, the pathophysiology beyond hyperglycaemic associated positive neurological manifestations, including seizures, remains to be elucidated. The occurrence of symptomatic focal epilepsy as a manifestation of diabetic related hyperglycaemia is seldom reported. Herein, we present a case of focal epilepsy with alternating positive and negative neurological manifestations as the initial manifestation of diabetes-related hyperglycaemia.

## 2 Case report

**Fig. 1:**
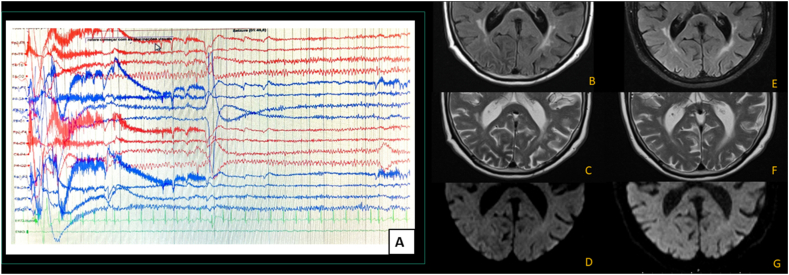
A. Ictal electroencephalogram showing occipital focal seizures following left oculo-cephalic version. There is associated right occipital diffuse cortical dysfunction. Initial brain magnetic resonance imaging (B,C,D) showing decreased signal intensity in Fluid-attenuated inversion recovery (B) and T2 (C) with increased cortical sign (D) on diffusion weighed imaging in the right middle occipital region. Follow up brain imaging (E,F,G) showing resolution of the brain signal abnormality.

An 81-year-old-man with type 2 diabetes mellitus, who was being treated with metformin, was brought to the emergency due to recurrent episodes of visual hallucinations lasting 1–2 minutes over the past 24 hours. He described this episodes as cloudy vision, elementary images consisting of triangles and coloured squares in his left visual field. These images would then evolve into complex images of people's faces and cars expanding to his right visual field. Neurological examination revealed the presence of left homonymous hemianopsia and right nasal hemianopia. He had abandoned the diabetic diet and markedly increased the consumption of sugar containing food and beverage in the previous days. Initial laboratory tests showed persistently high levels of glycemia (>350mg/dL) and traces of serum/urine ketones. During emergency electroencephalogram, two reflex seizures triggered by oculo-cephalic version were documented while the patient was experiencing visual manifestations, (Fig. 1A). Additionally, a focal decreased T2 signal intensity was documented in the right middle occipital region (Fig. 1B–D). Despite treatment with levetiracetam and lacosamide, seizures were only controlled with achievement of normoglycemia 48 hours after treatment with insulin and isotonic saline solution. He progressively improved and returned to his baseline status, with complete recovery of visual acuity and no campimetry defects, after 4 days of normoglycemia. Thentiepileptics were gradually discontinued. During follow-up, he remained free of seizures, and both the electroencephalogram and brain imaging (Fig. 1E–G) were unremarkable.

## 3 Discussion

The combination of two rare phenomena, positive (visual hallucinations) and negative (visual field amputation), that occurred in our patient is extremely rare. The transient visual field amputation is mostly likely an equivalent of Todd's phenomena following the occipital seizures. It appears that there is increased involvement of the posterior lobes in symptomatic seizures associated with hyperglycaemia [2]. One can speculate that as in the posterior reversible encephalopathy, the reduced vascular compliance of posterior circulation vessels may account for this finding [3]. Indeed, hyperviscosity in patients with severe hyperglycaemia may compromise cerebral circulation. Individual asymmetries in brain perfusion or specific localized brain functional or structural abnormalities may account for the focal nature of seizures [2]. In hyperglycaemic state, there is a shift away from the aerobic respiratory cycle (Krebs cycle) to an anaerobic pathway, leading to the depletion of the neuroinhibitory transmitter gamma aminobutyric acid (GABA) and causing a decrease in the seizure threshold [4]. Similar to some other cases [2], our patient exhibited transient brain focal structural changes. In general, there is a strong correlation between seizure-induced reversible signal changes in magnetic resonance and underlying brain structural changes, as well as ictal/interictal electroencephalogram findings [5]. This suggests that focal seizures may be triggered by localized subclinical structural brain abnormalities under an environment of decreased seizure threshold associated with hyperglycaemic status. Transient focal or diffuse central nervous system clinical imaging-electrophysiological abnormalities can occur in the context of systemic dehydration from different aetiologies [5]. Therefore, it is reasonable to consider that brain dehydration caused by hyperglycaemic status might mechanistically contribute to the occurrence of symptomatic focal epilepsy.

If an electroencephalogram had not been performed, the reflex focal seizure could have gone unnoticed. Reflex focal epilepsy is a rare condition in patients with hyperglycaemic states, making misdiagnosis highly possible. For instance, in our case, it could be misdiagnosed as a typical manifestation of acute metabolic encephalopathy. As reported in similar cases, seizures associated with hyperglycaemic status can be resistant to antiepileptics. It is important to consider the limitation of being a single case report, which of course hampers solid conclusions. Nevertheless, our case further validates that seizures associated with hyperglycaemic status can be resistant to antiepileptic treatment and only cease with glycaemic control [6].

The patient's written informed consent was obtained.

## Author contribution statement

All authors listed have significantly contributed to the investigation, development and writing of this article.

## Data availability statement

No data was used for the research described in the article.

## Declaration of competing interest

The authors declare that they have no known competing financial interests or personal relationships that could have appeared to influence the work reported in this paper.

## References

[1] Narayanan S. (2012). Hyperglycemia-induced Hemiballismus hemichorea: a case report and brief review of the literature. J. Emerg. Med..

[2] Wang C.P., Hsieh P.F., Chen C., Lin W.Y., Hu W.H., Yang D.Y., Chang M.H. (2005). Hyperglycemia with occipital seizures: images and visual evoked potentials. Epilepsia.

[3] Fugate J.E., Rabinstein A.A. (2015). Posterior reversible encephalopathy syndrome: clinical and radiological manifestations, pathophysiology, and outstanding questions. Lancet Neurol..

[4] Guisado R., Arieff A.I. (1975). Neurologic manifestations of diabetic comas: correlation with biochemical alterations in the brain. Metab. Clin. Exp..

[5] Mariajoseph F.P., Sagar P., Muthusamy S., Amukotuwa S., Seneviratne U. (2021). Seizure-induced reversible MRI abnormalities in status epilepticus: a systematic review. Seizure.

[6] Kalra S., Unnikrishnan A.G., Gupta Y. (2016). Epileptogenicity of diabetes and antiepileptogenicity of ketogenic states: clarity or confusion?. Indian J. Endocrinol. Metabol..

